# Shifting patterns in fine root distribution of four xerophytic species across soil structural gradients and years of growth

**DOI:** 10.1002/ece3.10889

**Published:** 2024-02-07

**Authors:** Hui Hu, Weikai Bao, Long Huang, Fanglan Li

**Affiliations:** ^1^ Chengdu Institute of Biology Chinese Academy of Sciences Chengdu Sichuan China; ^2^ Henan Key Laboratory of Water Pollution Control and Rehabilitation Henan University of Urban Construction Pingdingshan China

**Keywords:** arid ecosystem, fine root functional traits, root depth, rooting pattern, soil structure, temporal dynamics

## Abstract

Fine root (diameter < 2 mm) distribution influences the potential for resource acquisition in soil profiles, which defines how plants interact with local soil environments; however, a deep understanding of how fine root vertical distribution varies with soil structural variations and across growth years is lacking. We subjected four xerophytic species native to an arid valley of China, *Artemisia vestita*, *Bauhinia brachycarpa*, *Sophora davidii*, and *Cotinus szechuanensis*, to increasing rock fragment content (RFC) treatments (0%, 25%, 50%, and 75%, v v^−1^) in an arid environment and measured fine root vertical profiles over 4 years of growth. Fine root depth and biomass of woody species increased with increasing RFC, but the extent of increase declined with growth years. Increasing RFC also increased the degree of interannual decreases in fine root diameter. The limited supply of soil resources in coarse soils explained the increases in rooting depth and variations in the pattern of fine root profiles across RFC. Fine root depth and biomass of the non‐woody species (*A. vestita*) in soil profiles decreased with the increase in RFC and growth years, showing an opposite pattern from the other three woody species. Within woody species, the annual increase in fine root biomass varied with RFC, which led to large interannual differences in the patterns of fine root profiles. Younger or non‐woody plants were more susceptible to soil environmental changes than the older or woody plants. These results reveal the limitations of dry and rocky environments on the growth of different plants, with woody and non‐woody plants adjusting their root vertical distribution through opposite pathways to cope with resource constraints, which has management implications for degraded agroforest ecosystems.

## Graphical abstract

The increased extent of rooting depth in three woody species with increasing rock fragment content declined with plant growth years. Degrees of interannual decreases in fine root diameter of all species increased with increasing gravel content. The limited resource and deep penetration in high rocky soil led to deep fine root distribution.

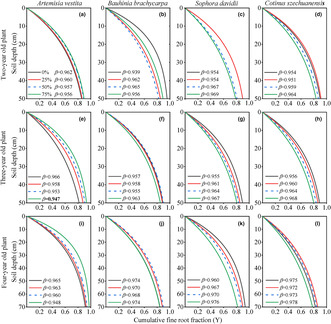

## 1 INTRODUCTION

The dimensions of rooting systems, indicated by root depth and lateral extent, represent the amount of soil space occupied, which determines the uptake of resources by fine roots (diameter < 2 mm) (Freschet et al., 2021; Schenk & Jackson, 2002; Zhou et al., 2020). Adjustment of root distribution in soil profiles is crucial for plant resource acquisition and can thus affect plant resilience to environmental stress (Fan et al., 2017; Zhou et al., 2020). For example, deep roots are a key strategy for xerophytes to survive in arid ecosystems (Fan et al., 2017; Schenk & Jackson, 2002; Zhou et al., 2020). Numerous studies have found that fine roots of woody plants are concentrated in topsoil (Gao et al., 2020; Gwenzi et al., 2011; Huang et al., 2008; Li et al., 2020; Yang et al., 2021; Zewdie et al., 2008), whereas roots distribution typically deepens in seasonally dry areas (Schenk & Jackson, 2005). The fine root vertical pattern of many plants exhibits high plasticity under soil heterogeneity (Li et al., 2020; Luo et al., 2021; Padilla & Pugnaire, 2007). Therefore, understanding the pattern of root distribution across environmental gradients may provide insight into the adaptive processes of plants under environmental stress.

Soil heterogeneity is caused by the patchy distribution of coarse particles and nutrients in soil profiles (Huang, Bao, et al., 2023; Huang, Hu, et al., 2023). Coarse parts of soil (particle size >2 mm), usually called rock fragments, are widespread in terrestrial ecosystems and their content plays an important role in soil hydrological conditions such as infiltration (Poesen & Lavee, 1994; van Wesemael et al., 2000; Zhang et al., 2011, 2018; Zhou et al., 2009). Many soils contain rock fragments as a result of processes of soil genesis and human activities such as tillage and resource extraction (Hu et al., 2021). Rock fragment content (RFC) is critical for other soil properties, such as soil physical structure (Gargiulo et al., 2015, 2016; Xu et al., 2012), water and nutrient availability (Ceacero et al., 2020; Rytter, 2012), and microbial composition (Hong et al., 2021; Huang, Bao, et al., 2023). In soils with high RFC, growth and biomass accumulation decreases in plants (Hu et al., 2021; Mi et al., 2016), but the mass fraction of the below‐ground parts increases (Hu et al., 2021). However, little is known about the vertical distribution pattern of fine root profiles in response to changes in RFC.

Plants adjust the soil space colonized by roots to obtain sufficient resources to cope with variations in the soil particle composition (Bengough, 2003; Schenk & Jackson, 2002, 2005). A strong sensitivity of rooting depth to local soil hydrological conditions has been identified (Fan et al., 2017; Schenk & Jackson, 2005; Zhou et al., 2020), whereby roots remain shallow in waterlogged land, but usually deepen in arid regions (Fan et al., 2017; Schenk & Jackson, 2002). Soil hydrology is determined by soil texture (Fan et al., 2017; Sperry & Hacke, 2002); coarse‐grained (sandy or gravel) soils with low water‐holding capacity allow deep infiltration profiles that encourage deep roots (Fan et al., 2017; Schenk & Jackson, 2005; Zhou et al., 2020). The coarse particles present in the soil also increase macropores and reduce mechanical resistance (Gargiulo et al., 2015, 2016; Xu et al., 2012), which is conducive to the penetration of the root system into the deep layer (Bengough, 2003; Clark et al., 2002). However, whether the rooting depth increases when the content of coarse soil particles increases and water and nutrient levels decrease remains to be explored.

In addition to response to environmental changes, the inherent characteristics of plants also determine root distribution and resource acquisition strategies, such as species and growth years (Gao et al., 2020; Luo et al., 2021; Peek et al., 2005; Zhou et al., 2020). Different species lead to varying root distribution and morphology, with woody plants having a deeper maximum root distribution than herbaceous plants (Li et al., 2020; Yang et al., 2021). However, it is not yet known how the root spatial distribution of different species responds to soil particle composition changes, and whether there are differences. Simultaneously, stand age as a key inherent characteristic also determines biomass and spatial distribution of roots (Peek et al., 2005; Peichl & Arain, 2006; Zhang et al., 2018). Previous studies have focused on the seasonal dynamics of fine root distribution in the short term (Cheng & Bledsoe, 2002; O'Grady et al., 2005; Wang et al., 2016), this seriously limits the understanding of the dynamic changes in root distribution of plants at different growth years and their time cumulative responses to soil heterogeneity. In addition, compared to short‐term or one‐time sampling, studies of long‐term and continuous growth years can better reflect the response strategies in plant roots to changing external environments.

In the present study, we observed the fine root vertical profiles of four xerophytic species along an RFC gradient (0%, 25%, 50%, and 75%, v v^−1^) and during 3 years of growth (the second, third, and fourth years) in the arid valley environment of western China (Hu et al., 2021). These four species are important native species in the arid valleys of the Hengduan Mountain region, with irreplaceable ecological value in vegetation restoration and soil and water conservation (Bao et al., 2012; Li, Bao & Wu, 2008; Li, Bao, Wu & You, 2008; Wu et al., 2008). For microhabitat heterogeneity, differences in plant adaptations are recognized based on aboveground measurements (Hu et al., 2021; Li, Bao, Wu & You, 2008; Wu et al., 2008), whereas the variation in rooting profile and its ecological indicator is not well known. We examined fine root biomass and morphology across the soil layers along the RFC gradient for three years of growth. Our objectives were to investigate: (1) how the vertical pattern of fine root depth and traits varied with the RFC gradient, (2) the temporal pattern of fine root depth and traits along soil profiles and RFC, and (3) interspecific differences in the dynamic response of fine root depth and vertical distribution to the RFC gradient. We hypothesized that: (1) root distribution deepened with the increase in RFC, based on the characteristics of poor water‐holding capacity and low strength of coarse soils (Bengough, 2003; Fan et al., 2017; Schenk & Jackson, 2002, 2005); (2) with the increase in growth years, the response of root distribution to changes in RFC showed a cumulative decreasing effect of time, because younger plants (seedlings) are more sensitive to soil heterogeneity than older ones (Padilla & Pugnaire, 2007).

## 2 MATERIALS AND METHODS

### 2.1 Study site

The study site is located at Jingzhou Hill, Maoxian County, in the arid valley of the Minjiang River, Sichuan, China (31°70′ N, 103°87′ E, altitude 1637 m). The RFC ranges from 1% to 65% (g g^−1^), and most rock fragments have a particle size larger than 10 mm (Bao et al., 2012). These characteristics served as the basis for the design of the RFC gradient and selection of particle size. The mean annual precipitation at the nearest climate station (Maoxian County Meteorological Station, 2 km from the study site) was 495 mm, with 83% of the precipitation occurring during the growing season from May to October. The mean annual potential evaporation is 1332 mm, and the annual air temperature is 15.6°C. The site has historically been cultivated for agricultural crops, with potatoes and celery planted 2 years before the start of the present study. Typically, cinnamon soil has a clay loam with coarse texture and low fertility (Bao et al., 2012). The soil depth is typically 50–70 cm.

### 2.2 Experimental design

Our study was conducted under semi‐controlled (control the proportion of soil and rock fragments without controlling climate conditions) field conditions over 4 years in the arid valley of the Minjiang River. The experiment used a randomized block design with 16 treatments as combinations of four plant species and four RFC (0%, 25%, 50%, and 75% volumetric content, v v^−1^). Each treatment had three replicates (plots), making a total of 48 plots (4 RFC × 4 species × 3 replicates) in the study. The four xerophytic species evaluated were *Artemisia vestita*, *Bauhinia brachycarpa*, *Sophora davidii*, and *Cotinus szechuanensis*, which are native to the arid valley. They were chosen because of their ecological importance and divergent root performance: *A. vestita*, a fast‐growing perennial herbaceous plant (half‐shrubby) with shallow root distribution; *B. brachycarpa*, a non‐nitrogen‐fixing legume shrub with a thin and high branching root system (root branching density = 1.47 No. cm^−1^); *S. davidii*, a nitrogen‐fixing legume shrub with thick and low branching root system (root branching density = 1.07 No. cm^−1^); and *C. szechuanensis*, a tall shrub with developed root hairs and mycorrhiza. The four species all begin to reproduce in the second year, while three shrubs have a lifespan over 50 years and *A. vestita* has a lifespan of several decades.

Each plot was a pit, with dimensions of 1 m × 1 m × 0.5 m (length × width × depth) and 50 cm spacing between plots. The soil at a depth of 0–50 cm was excavated from each pit. Subsequently, the walls of each pit were lined with polyethylene film to prevent interference from external conditions, and the bottom of the plot was left unlined to allow natural drainage. Fine soil particles (<2 mm in diameter) and rock fragments (10–20 mm in diameter) were collected, mixed uniformly, and filled back into each pit to obtain the desired RFC.

Soil from all pits was then air dried for a week, and fine soil particles were collected by passing the dried soil through a 2 mm sieve and all the soil was then mixed uniformly. Initial soil properties in the samples (*n* = 6) were as follows: total carbon, 15.3 ± 0.09 g kg^−1^; total nitrogen, 2.31 ± 0.02 g kg^−1^; and total phosphorus, 0.61 ± 0.01 g kg^−1^. Thin‐bedded limestone (dominated by phyllite), which is commonly found in regional soil, was used for the rock fragments in this study. A sufficient amount of rock fragments was collected from the coarse soil part (≥2 mm) that had not passed through the sieve and from nearby land. The crushed rock materials were first passed through a 10 mm sieve and then a 20 mm sieve, leaving rock fragments with a particle size of 10–20 mm for use. The density of thin‐bedded limestone was 2.56 ± 0.03 g cm^−3^ (*n* = 12), as measured by the water displacement method (Wang et al., 2017). After uniform mixing, fine soil particles and rock fragments were filled back into each pit at the desired RFC in April 2018. Each plot was irrigated with 100 L of water and left for soil to stabilize.

Sowing in each pit was performed at a depth of 0.5–1 cm with an equidistant interval pattern of nine points (25 cm equidistance between two points) in April 2018 (Hu et al., 2021). Seeds of the four species were collected from their natural habitats in the arid valley of the Minjiang River (31°42′ N, 103°53′ E, altitude range of 1600–1920 m) from August to October 2017, air‐dried for 4–8 days, and stored at room temperature (10–25°C). All the seeds were disinfected by immersion in 2.5% NaClO for 1 h and then sown in each plot. Seedlings were watered weekly within 1 month after sprouting to prevent early losses. They were thinned 2 months after sprouting (Hu et al., 2021), leaving four average‐sized seedlings (seedlings with moderate plant height and diameter under the same species and treatment) per plot and an interval of approximately 50 cm between seedlings. The plots were weeded (the weeds were pulled up by hand) twice a week to ensure normal plant growth.

### 2.3 Measurements

#### 2.3.1 Root and soil sampling

The average plant, which is a standard plant with the closest plant height and basal diameter to the average value under the same species and treatment, was selected in each plot for sampling roots in late September 2019, 2020, and 2021, representing plant growth for the second, third, and fourth years, respectively. Soil cores (7 cm inner diameter) were used to sample root segments and soil from vertical soil profiles. In each plot, two cores were extracted at 10 cm from the plant base in different directions. Each soil core was sampled to a depth of 50 cm and divided into five layers (0–10, 10–20, 20–30, 30–40, and 40–50 cm) in the second and third years because the maximum distribution depth of fine roots in most plants does not exceed 50 cm in these two years. Sampling of the 50–70 cm soil layer was performed in the fourth year. The root segments and soil of each soil layer from the two cores were mixed into one sample in each plot to estimate the root morphology and dry weight.

#### 2.3.2 Vertical distribution

After separating root segments from the soil, root samples were separated into coarse and fine roots (<2 mm in diameter). Fine root samples were carefully washed with deionized water and scanned using a root image scanner (Epson V800; Seiko Epson Corp., Japan). The average diameter, total length, and total volume of fine roots in each plot were determined using the software Win‐RHIZO 2020 (Regent Instruments, Canada). The scanned fine roots were oven‐dried at 65°C for 48 h and then weighed. Finally, the biomass, length density, specific length, and tissue density of fine roots were calculated using the following formulas:

$$\text{Fine root biomass}\;\left(g\;m^{-2}\right)=\text{Fine root}\;\mathrm{dry}\;\text{mass}\;\left(g\right)/\text{Soil core sectional area}\;\left(m^{2}\right)$$

$$\text{Fine root length density}\;\left(\mathrm{cm}\;{\mathrm{cm}}^{-3}\right)=\text{Fine root length}\;\left(\mathrm{cm}\right)/\text{Soil core volume}\;\left({\mathrm{cm}}^{3}\right)$$

$$\text{Specific fine root length}\;\left(m\;g^{-1}\right)=\text{Fine root length}\;\left(m\right)/\text{Fine root}\;\mathrm{dry}\;\text{mass}\;\left(g\right)$$

$$\text{Tissue density}\;\left(g\;{\mathrm{cm}}^{-3}\right)=\text{Fine root}\;\mathrm{dry}\;\text{mass}\;\left(g\right)/\text{Fine root volume}\;\left({\mathrm{cm}}^{3}\right)$$

The rooting depth is indicated by *β* value. *β* is a simple numerical index of the rooting distribution based on the asymptotic equation:

$$Y=1–\beta^{d}$$

where *d* is the soil depth (cm), *Y* is the cumulative root biomass fraction from the surface to a soil depth of *d* (*Y*(*d*) = cumulative root biomass of 0–d cm/cumulative root biomass of 0–50 or 70 cm), and *β* is the fitted coefficient. Low *β* values correspond to shallow root allocation, whereas high *β* values correspond to an increased proportion of roots with depth.

#### 2.3.3 Soil properties

Soil was also sampled from soil cores along the vertical soil profile (see section 2.3.1). Soil samples from each layer were divided into two subsamples. Fresh subsamples were immediately weighed and oven‐dried at 105°C to determine the soil water content (SWC, g g^−1^). The remaining soil subsamples were air‐dried and passed through a 100‐mesh (0.15 mm) sieve to determine soil physicochemical properties. Soil total carbon (TC) and total nitrogen (TN) levels were determined by combustion in an elemental analyzer (Vario MAX; Elementar, Germany), and total phosphorus (TP) was measured using the sulfuric acid‐soluble perchlorate acid‐molybdenum antimony colorimetric method (Hu et al., 2016). Dissolved organic carbon was extracted with deionized water (1:5 w/v soil to water) and determined using an elemental analyzer (Vario Macro Analyzer; Elementar, Germany). Ammonium nitrogen (NH_4_^+^‐N) and nitrate nitrogen (NO_3_^−^‐N) were extracted in the 2 mol L^−1^ KCl solution, and the supernatant was measured using a flow analyzer (SEAL Analytical, Germany). Available phosphorus (aP) was extracted with 0.5 mol L^−1^ NaHCO_3_ at pH 8.5, and then determined by molybdenum antimony anti‐colorimetry. Soil pH was determined in a 1:2.5 (w/v) soil‐water suspension (SevenEasyS20; Mettler Toledo, USA) (Huang, Hu, et al., 2023). Due to the long experimental design period (5–6 years) and funding constraints, all soil properties will only be measured in the second and third years.

### 2.4 Statistical analyses

Three‐way analysis of variance (ANOVA) was used to examine the effects of RFC, year of growth, plant species, and their interactions on fine root traits. Average root traits of entire soil profiles were used to calculate the percentage variation between rock‐free soil and RFC treatments, as well as between the second year and other years. Thus, the variation range between RFC treatments and years of growth could be clearly indicated by points and lines. One‐way ANOVA was used to determine significant differences in *β*, biomass, length density, specific length, diameter, and tissue density of fine roots between RFC treatments. Data meeting the assumption of homogeneity of variance were tested by least significant difference (LSD); otherwise, data were analyzed using a non‐parametric test (Kruskal–Wallis). The relationships between fine root traits and soil properties in the soil profile were tested using linear fitting. Principal component analysis was performed using the mean values of fine root traits on the entire soil profile and *β* to obtain an overview of the multidimensional root functions of different years of growth and species. We also performed redundancy analysis using fine root traits and soil properties in each soil layer of the second and third years along the RFC gradient. Data were analyzed using SPSS 25.0 (IBM, USA) for means and ANOVA. The *β* coefficient, line graph, and histogram were generated using the software Origin 2018 (OriginLab Corporation, USA). Principal component and redundancy analyses were implemented using the package “vegan” in R software (version 4.0.4; R Core Team, Austria).

## 3 RESULTS

### 3.1 Rooting depth with different RFC treatments

**FIGURE 1:**
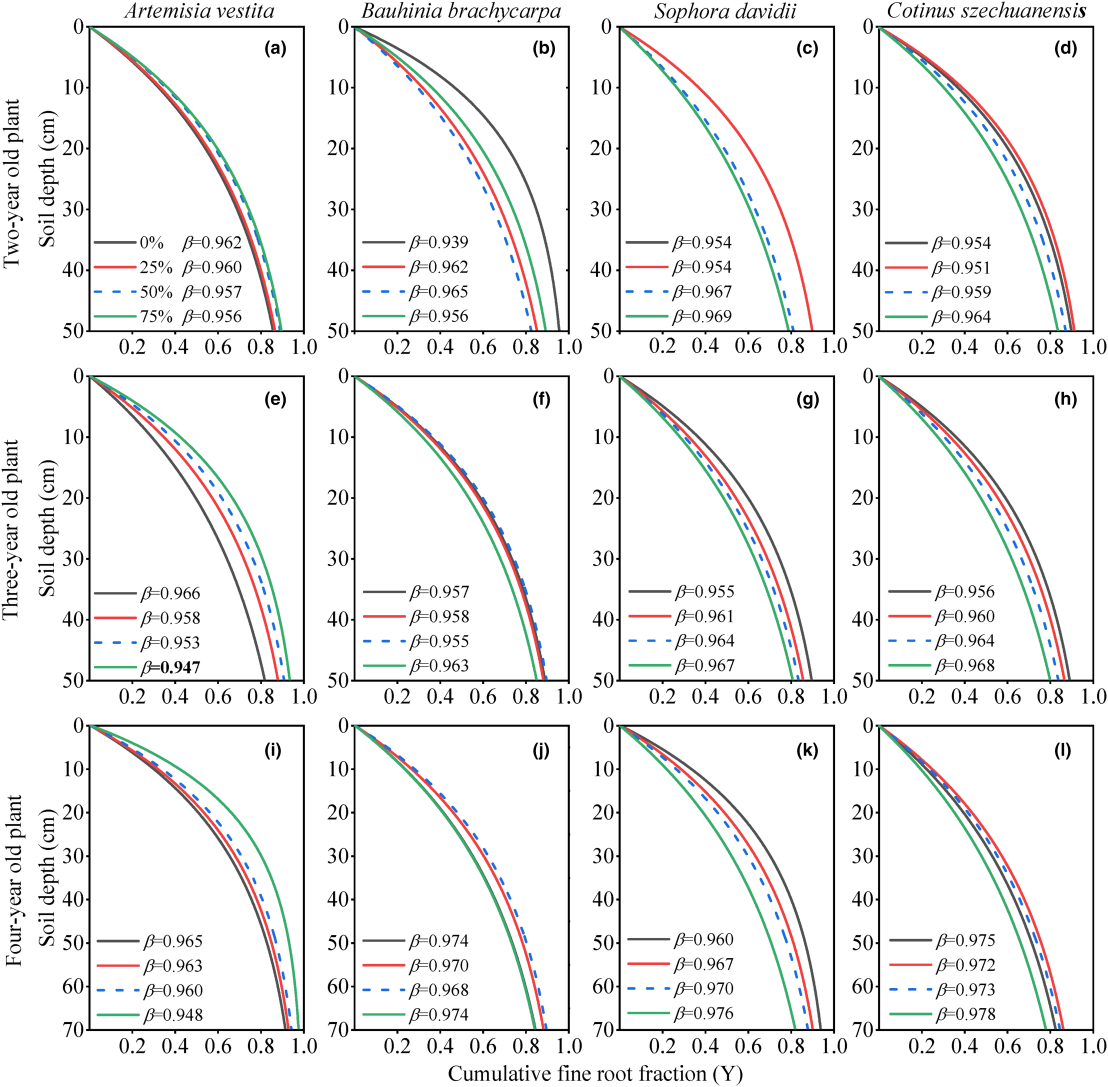
Models of vertical fine root distribution in each species across different soil rock fragment content (0%, 25%, 50%, and 75%) and years of growth. The curve of each rock fragment content was derived from the *β* parameter (*p* < .05 for all cases and did not show). *β* was estimated by 3 repetitions as the following function: *Y* = 1−*β* ^*d*^, where the cumulative root fraction of fine root (*Y*) from the surface to any depth (*d*), *Y*(*d*) = cumulative root biomass of 0–*d* cm/cumulative root biomass of 0–50 or 70 cm. Larger values of *β* indicate deeper rooting profiles.

**FIGURE 2:**
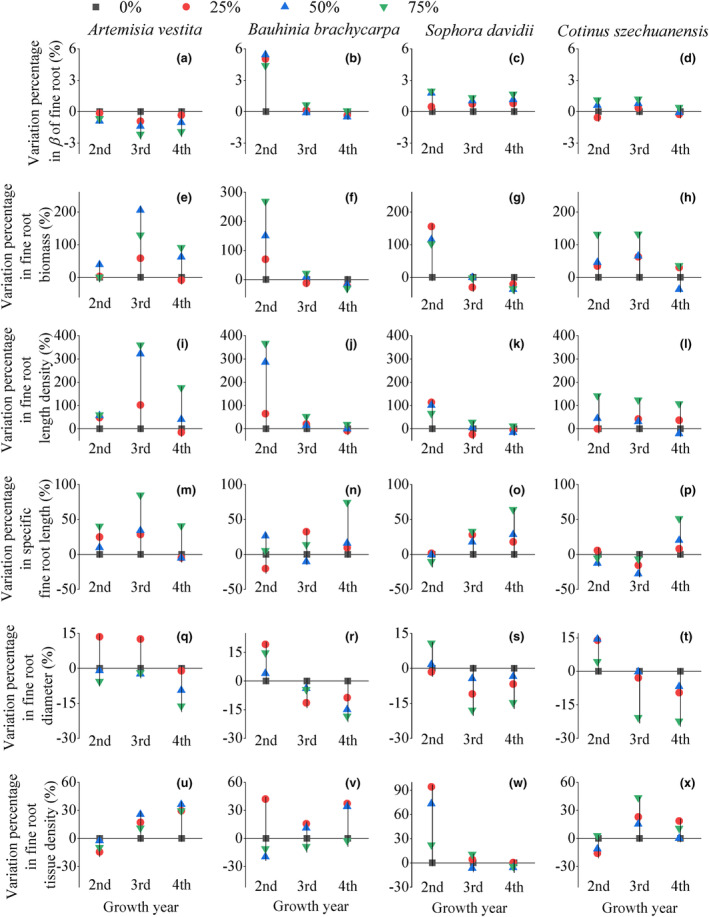
Variation range in *β*, biomass, and morphology traits of fine root in each species between different levels of soil rock fragment content (indicated by line). Point indicated variation percentage from 0% to 25%, 50%, and 75% RFCs. Fine root traits were mean value of whole soil depth in each growth year.

The fine root depth coefficient (*β*) of the three woody species increased with the increase in RFC gradient, and these species exhibited the deepest distribution at 75% RFC over the 3 years (Figures 1 and 2). However, the rooting pattern of fine roots in *A. vestita* was contrary to that of the other woody species along the RFC gradient (*p* < .01, Table A1). The *β* of *A. vestita* decreased with increasing RFC and was the largest in rock‐free soil over the 3 years (Figures 1 and 2a). With the increase in growth years, the variation range of fine root depth along the RFC gradient showed an increasing trend in *A. vestita* (Figure 2a–d) but showed a decreasing pattern for the three woody species (Figure 2).

### 3.2 Vertical distribution of fine roots with different RFC treatments

**FIGURE 3:**
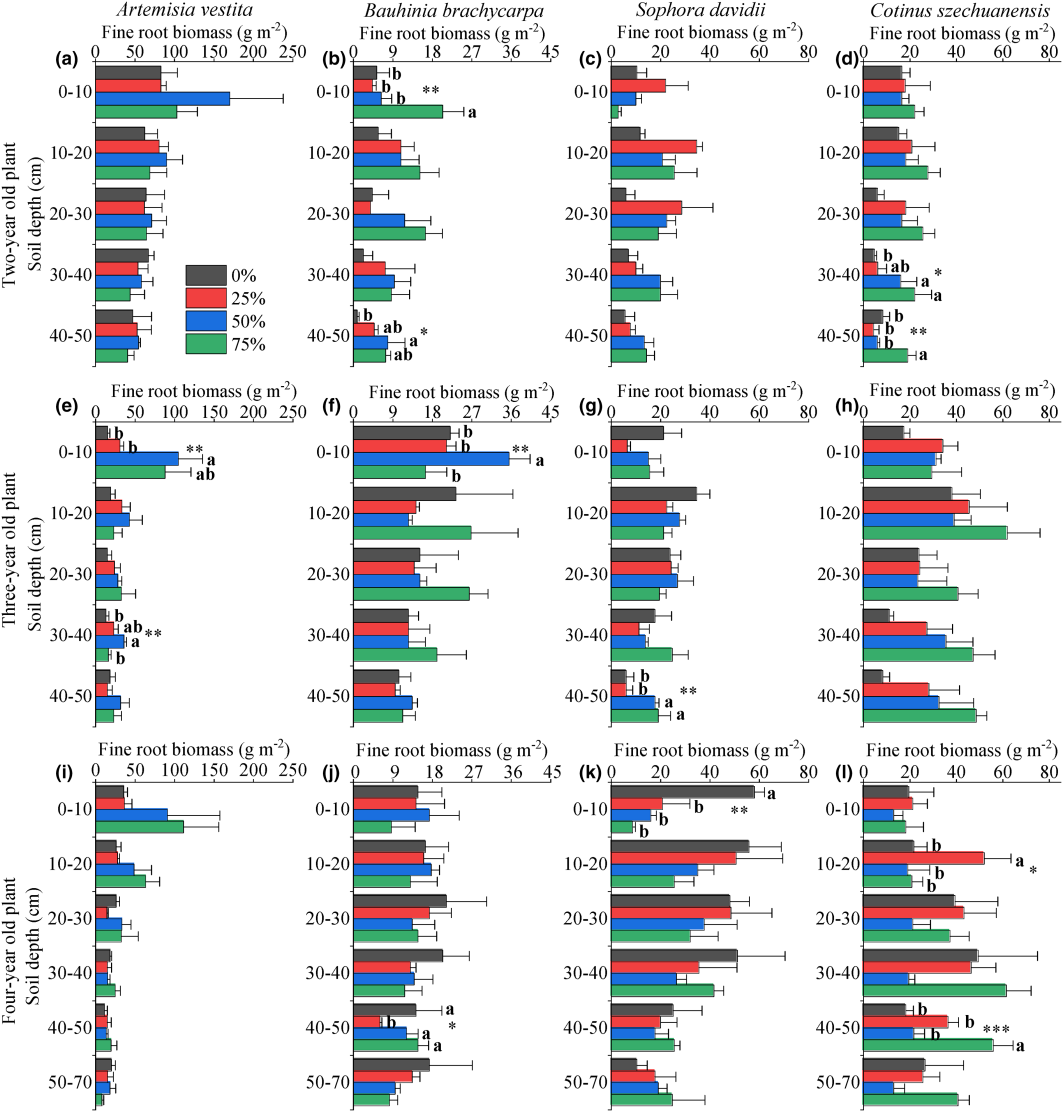
Fine root biomass of each species at different soil depths along four soil rock fragment content (0%, 25%, 50%, and 75%) and three years of growth. Bars represent means ± SE; *n* = 3. Different lowercase letters indicate significant differences between rock fragment content levels, *, **, and *** designate differences at *p* < .1, *p* < .05, and *p* < .01, respectively.

**FIGURE A1:**
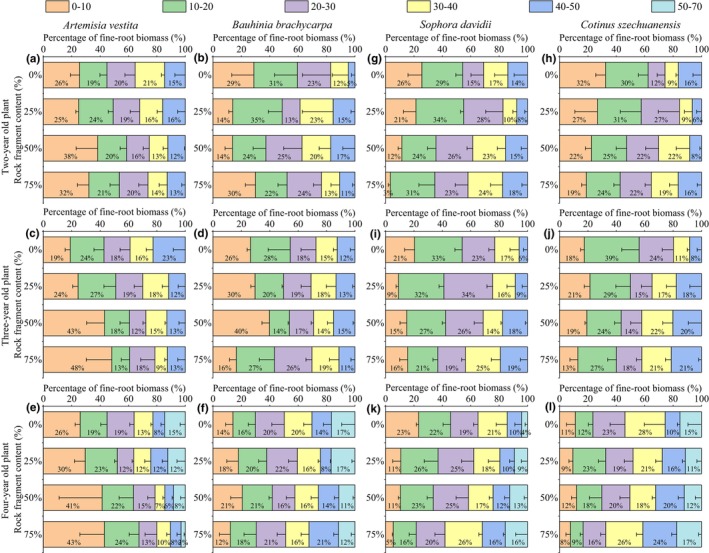
Percentage of fine root biomass in each species at different soil depths along four soil rock fragment content (0%, 25%, 50%, and 75%) and three years of growth. Bars represent means ± SE; *n* = 3.

Plants generally distributed a large proportion of fine roots in the soil layer above 30 cm (more than 60%, Figures 3 and A1). The vertical distribution of fine root biomass varied significantly between the different RFC treatments and species (*p* < .01, Table A1). Fine root biomass of woody species increased in most soil layers at 50–75% of RFC, and in particular, biomass increased in soil depth below 30 cm (Figures 3 and A1). Along the RFC gradient, the increasing range of average fine root biomass in woody species was the greatest in the first year (Figure 2). In 50–75% of RFC, the fine roots of *A. vestita* were concentrated in the surface layer, whereas those of the other three woody species were distributed in deep soils (Figures 3 and A1).

**FIGURE A2:**
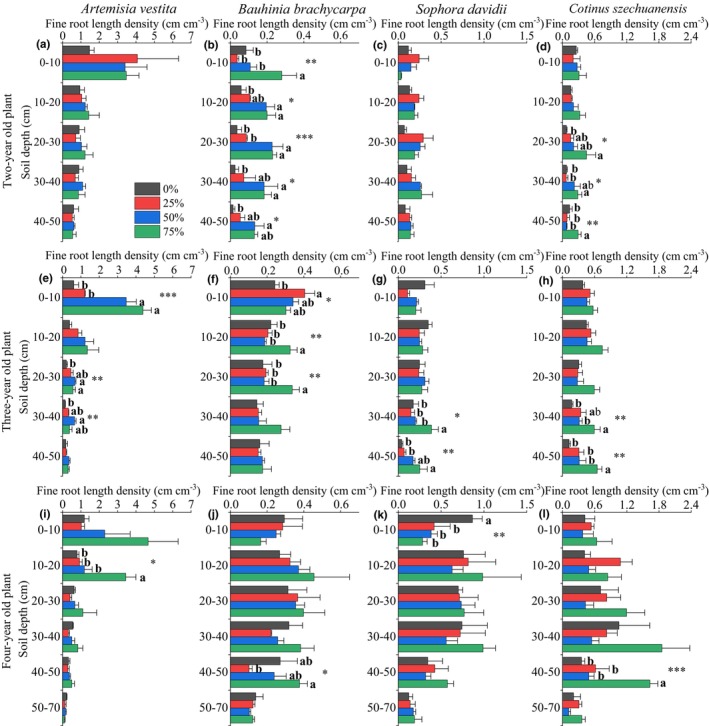
Fine root length density of each species at each soil depth across different soil rock fragment content (0%, 25%, 50%, and 75%) and years of growth. Bars represent means ± SE; *n* = 3. Different lowercase letters indicate significant differences between rock fragment content levels, *, **, and *** designate differences at *p* < .1, *p* < .05, and *p* < .01, respectively.

**FIGURE A3:**
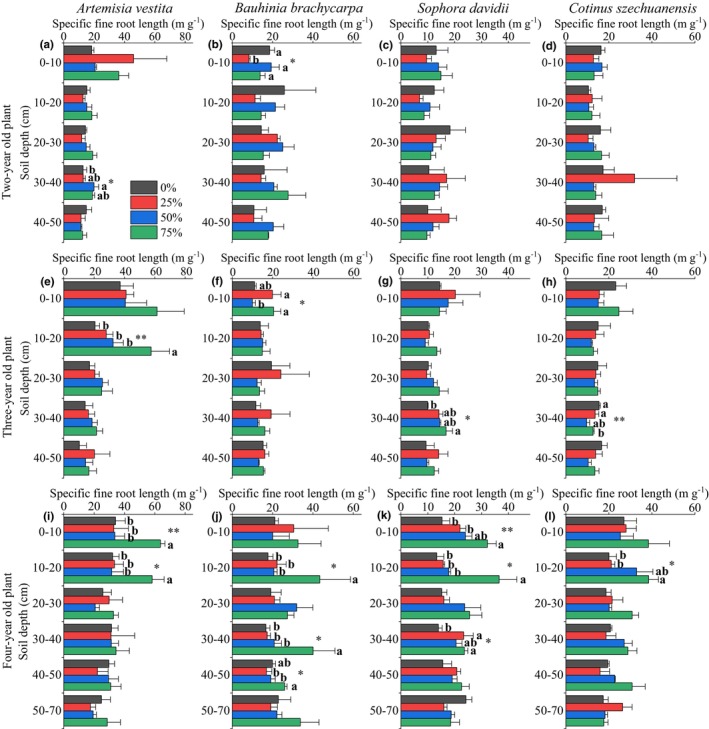
Specific fine root length of each species at different soil depths along four soil rock fragment content (0%, 25%, 50%, and 75%) and years of growth. Bars represent means ± SE; *n* = 3. Different lowercase letters indicate significant differences between rock fragment content levels, *, **, and *** designate differences at *p* < .1, *p* < .05, and *p* < .01, respectively.

**FIGURE A4:**
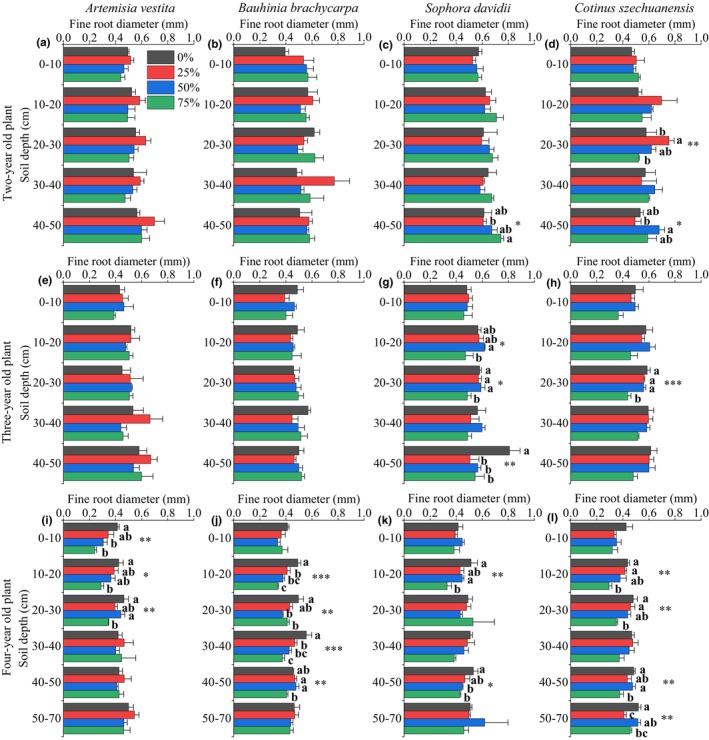
Fine root diameter of each species at different soil depths along four soil rock fragment content (0%, 25%, 50%, and 75%) and years of growth. Bars represent means ± SE; *n* = 3. Different lowercase letters indicate significant differences between rock fragment content levels, *, **, and *** designate differences at *p* < .1, *p* < .05, and *p* < .01, respectively.

**FIGURE A5:**
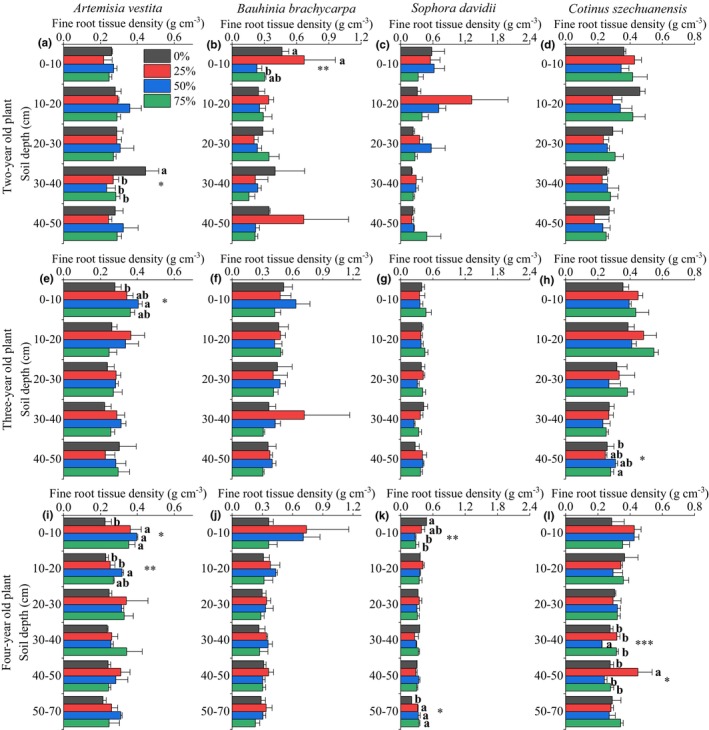
Fine root tissue density of each species at different soil depths along four soil rock fragment content (0%, 25%, 50%, and 75%) and years of growth. Bars represent means ± SE; *n* = 3. Different lowercase letters indicate significant differences between rock fragment content levels, *, **, and *** designate differences at *p* < .1, *p* < .05, and *p* < .01, respectively.

Vertical patterns of fine root length density among RFC treatments, years, and species were similar to those of fine root biomass (Figures 2, 3, and A2). The fine root–specific length, diameter, and tissue density of the four species did not show distinct changes with varying soil profiles (Figures A3, A4, A5). With the increase in RFC, the average fine root–specific length and tissue density increased in all species; however, the average diameter decreased (Figure 2).

### 3.3 Temporal dynamics of fine root distribution with growth

**FIGURE 4:**
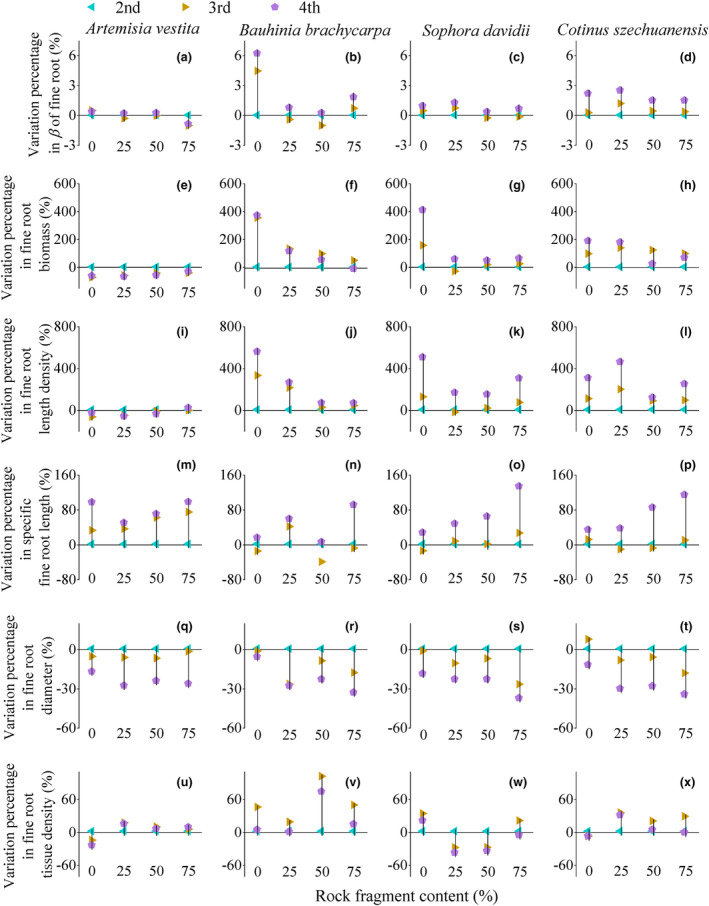
Variation range in *β*, biomass, and morphology traits of fine root in each species between three years of growth (indicated by line). Point indicated variation percentage from the second to the third, and the fourth year. Fine root traits were mean value of whole soil depth in each growth year.

**FIGURE 5:**
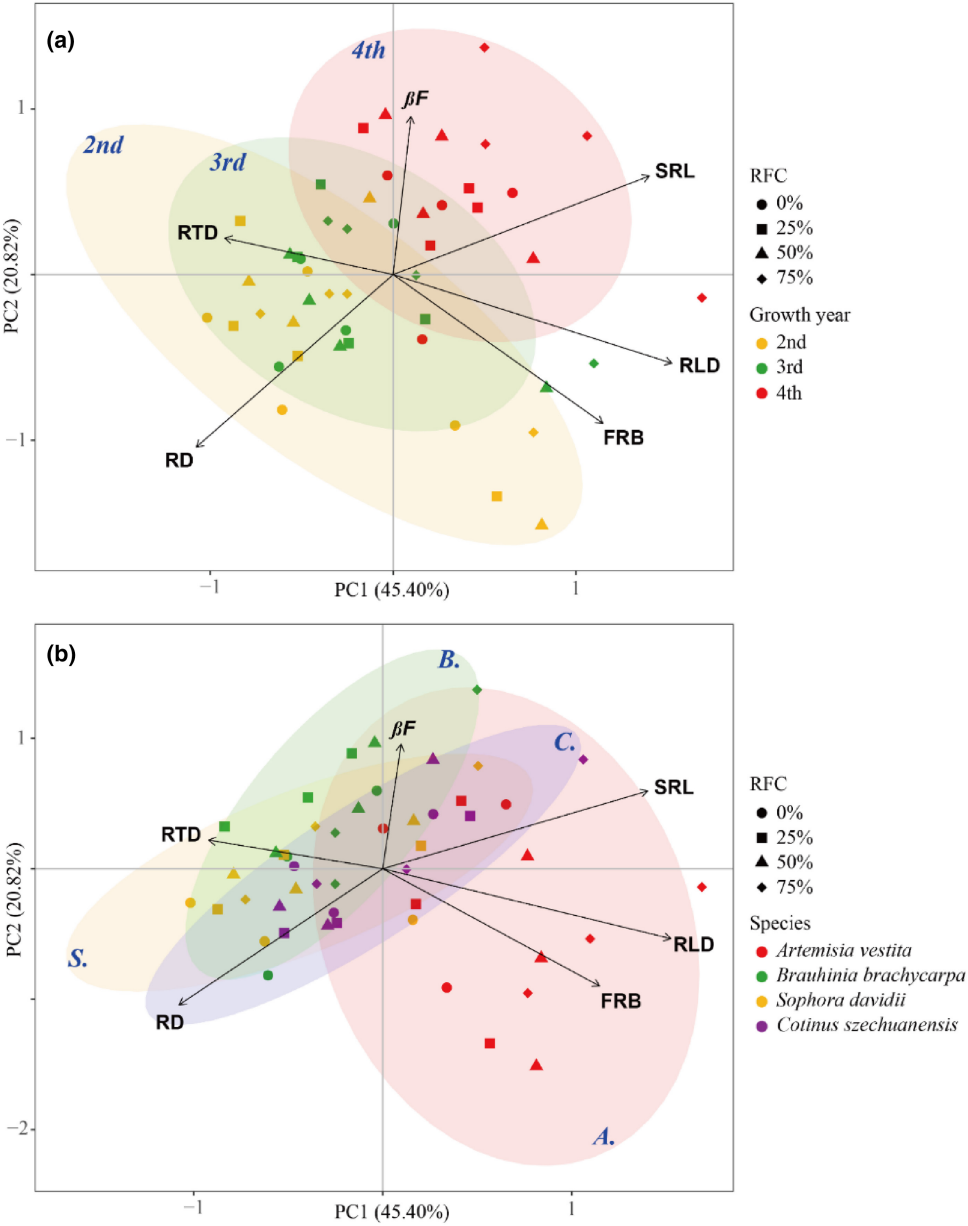
Principal component analysis of fine root traits (means of total soil layers) of the four species along rock fragment content gradient and years of growth. The proportions explained of Axis 1 and Axis 2 are 45.40% and 20.82%, respectively. Solid black lines indicate six fine root functional traits. The confidence circle represents the 95% confidence interval between growth years (a), yellow is second, the green is third, and the red is fourth. The confidence circle represents the 95% confidence interval between species (b), the red is *A*. (*Artemisia vestita*), the green is *B*. (*Bauhinia brachycarpa*), the yellow is *S*. (*Sophora davidii*), and the blue is *C*. (*Cotinus szechuanensis*). Abbreviations for root traits are as follows: *βF*, *β* of fine root; FRB, fine root biomass; RD, fine root diameter; RLD, fine root length density; RTD, fine root tissue density; SRL, specific fine root length.

The fine root distribution patterns in the soil profiles did not change with years of growth (Figures 3 and A2, A3, A4, A5); however, the depth and mean values of fine root traits in the entire soil profile changed considerably (Figures 4 and 5). Rooting depth, biomass, and length density of fine roots in the three woody species increased with increasing years, and the range of increase declined with increasing RFC (Figure 4). However, these parameters in *A. vestita* decreased with increasing years (Figures 3, 4, and A1). For all species, fine root–specific length increased and its diameter decreased with increase in years for all RFC levels (Figures 4 and 5), and the interannual variations were maximum under 75% RFC (Figure 4).

### 3.4 Relationship between root traits and soil properties

**FIGURE 6:**
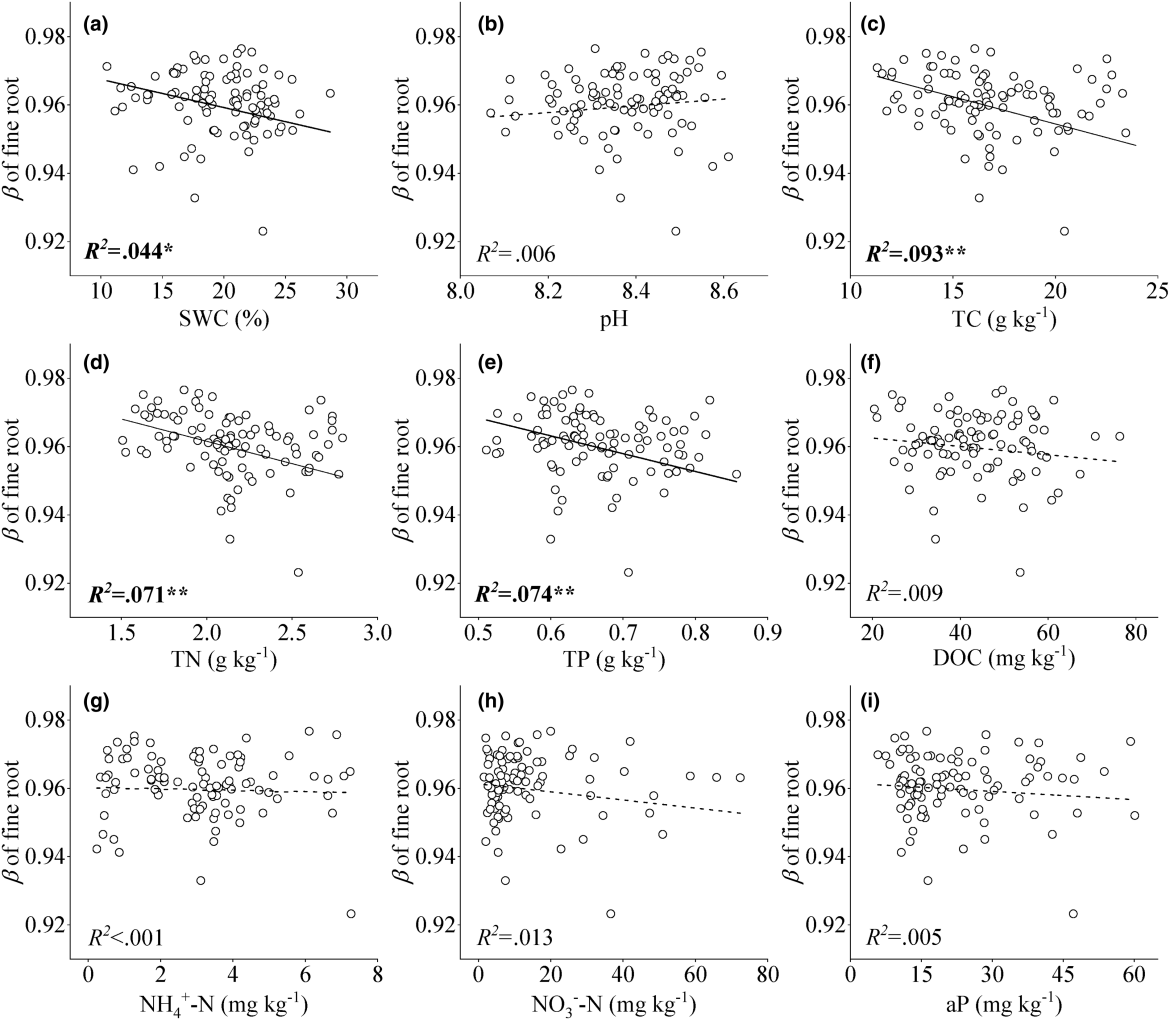
Relationships between fine root trait and soil properties in soil profile across years of growth and along rock fragment content gradient.

**FIGURE 7:**
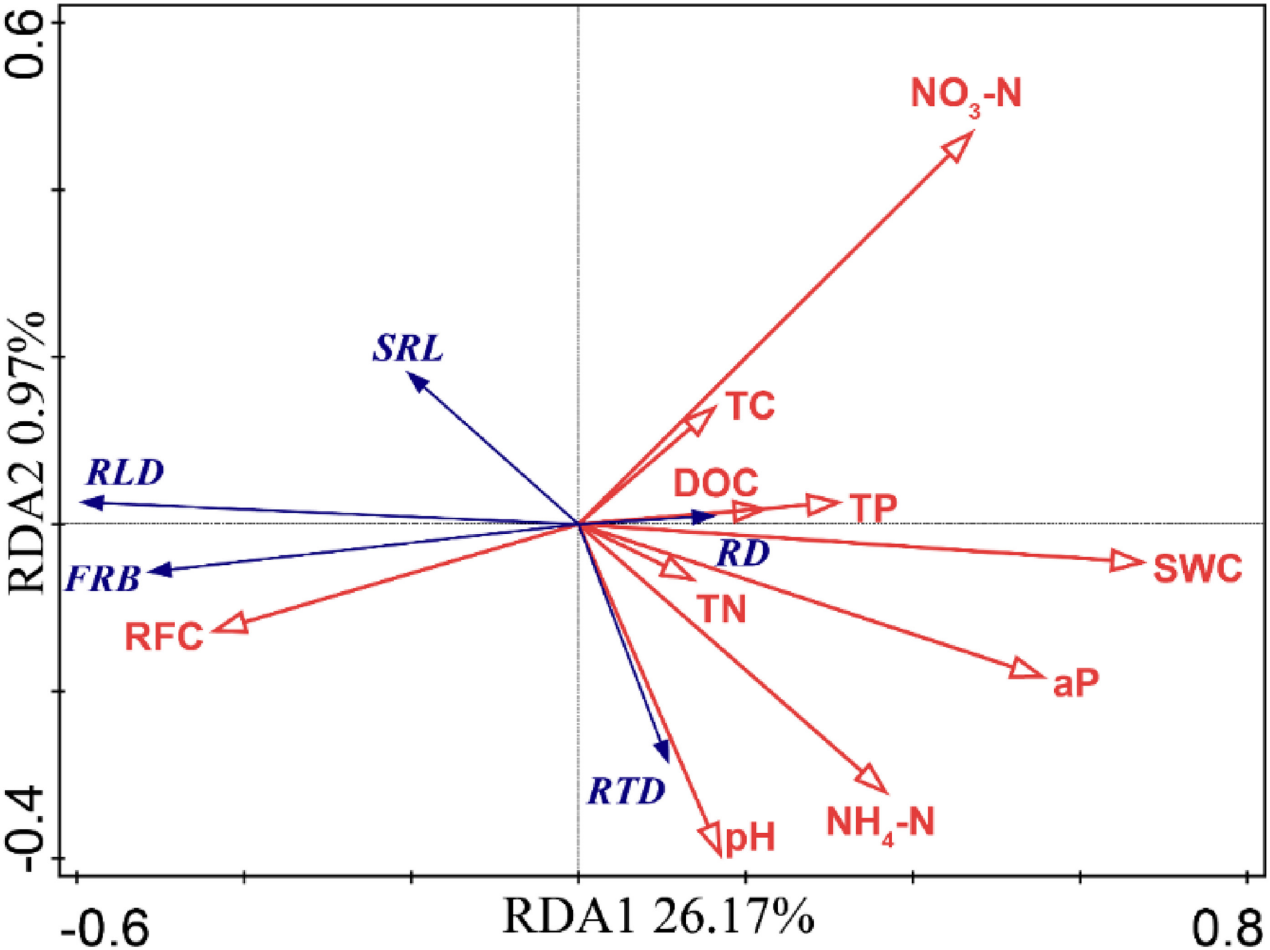
Redundancy analysis of fine root traits and soil properties along rock fragment content gradient in the second and third year. The proportions explained of Axis 1 and Axis 2 are 26.17% and 0.97%, respectively. Solid blue lines indicate fine root traits in each soil depth. Solid red lines indicate soil properties in each soil depth. Abbreviations for root traits are as follows: aP, soil available phosphorus; DOC, soil soluble carbon; FRB, fine root biomass; NH_4_‐N, soil ammonium nitrogen; NO_3_‐N, soil nitrate nitrogen; RD, fine root diameter; RFC, rock fragment content; RLD, fine root length density; RTD, fine root tissue density; SRL, specific fine root length; SWC, soil water content; TC, total soil carbon; TN, total soil nitrogen; TP, total soil phosphorus.

**FIGURE A6:**
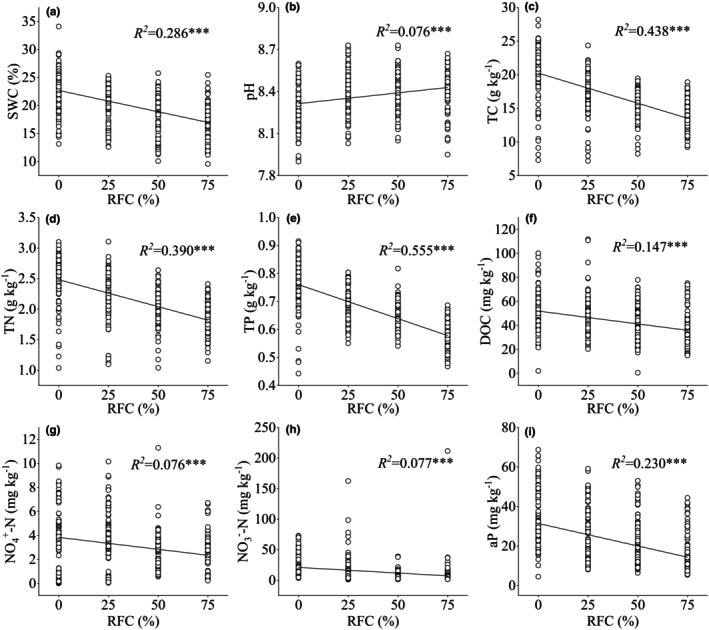
Variation trend of soil properties in the second and third year along rock fragment content (0%, 25%, 50%, and 75%).

The rooting depth of the four species was negatively correlated with SWC and nutrient content along the RFC gradient (*p* < .05, Figure 6). Reductions in soil water, TC, TN and TP were the main factors influencing the depth of the fine root profiles under high RFC conditions (Figures 6 and A6). Variations in SWC along the RFC gradient was the main explanatory factor for changes in fine root traits across soil profiles (Figure 7 and Table A2). Fine root biomass, length density, and specific root length all showed an increasing trend with the increase in RFC and were significantly correlated with the decrease in both SWC and available phosphorus (Figure 7).

## 4 DISCUSSION

Our findings partly support the first hypothesis that the vertical depth of fine roots in woody species increases with increasing RFC. Plants distributed more than 60% of fine root biomass in a soil depth above 30 cm and their presence reduced exponentially with soil depth. Fine root biomass and length density increased with increasing RFC in most soil layers, and this increase was highly noticeable in soil layers below 30 cm. The increase in fine root depth, average biomass, and length density range of woody species with increasing RFC were maximum in the second year, but minimum in the fourth year, which is consistent with our second hypothesis. Fine root diameter in all species decreased with increasing growth years, and the thinnest roots were found in soil profiles with 75% RFC. We also found that fine root depth, biomass, and length density of *A. vestita* decreased with increasing RFC and growth years, whereas the other three woody species presented maximum values under 75% RFC and in the last year.

### 4.1 Fine root depth increased with RFC

The potential fine root depth (*β*) in woody species increased with increasing RFC gradient over 3 years (Figures 1 and 2). Fine root biomass below a soil depth of 30 cm increased noticeably at high RFC (Figure 3); these adjustments of below‐ground biomass in vertical profiles caused the *β* to increase along the RFC gradient. A deep root distribution implies that plants occupy increased vertical space in the soil and have a better chance of accessing adequate resources in soils with high RFC (Freschet et al., 2021; Jackson et al., 2000; Padilla & Pugnaire, 2007; Zhou et al., 2020). The coarse texture of the rocky soil resulted in rapid infiltration, which led to deep water infiltration in the soil profile (Fan et al., 2017; Schenk & Jackson, 2002, 2005). Plants typically extend the vertical distribution of roots to follow deep infiltration in coarse soils (Fan et al., 2017; Laio et al., 2006; Schenk & Jackson, 2002, 2005). In addition, the increase in RFC also led to a decrease in water and nutrient levels (Figures 3 and A6), which encouraged plants to increase root depth and biomass in the soil profiles to ensure resource access (Figure 6; Jackson et al., 2000; Li et al., 2020; Padilla & Pugnaire, 2007; Zhou et al., 2020).

Across the soil profiles, both the length density and specific length of fine roots increased in all observed species with increasing RFC (Figures 2 and 7). Increases in length density and specific length of fine roots in soil profiles can promote the exploration range of roots in soil and facilitate root access to limited soil water and nutrients in high RFC environments (Comas & Eissenstat, 2009; Craine & Dybzinski, 2013; Freschet et al., 2018, 2021). Soils with high RFC also showed an overall decrease in fine root diameter (Figures 2, 7, and A4). This was because soil macroporosity increases with increasing RFC (Huang, Hu, et al., 2023), which reduces the soil mechanical resistance (Gargiulo et al., 2016; Xu et al., 2012) and results in finer root systems (Bengough, 2003; Clark et al., 2003). Finer roots are beneficial for root‐soil contact and resource acquisition in barren soil with high gravel content (Figure 7; Bengough, 2003; Freschet et al., 2020; Ma et al., 2018).

### 4.2 Fine root vertical profile varied with growth years

An important finding of this study is that with increasing growth, the changes in soil structures have a cumulative decreasing effect on the fine root vertical distribution. Over the years, the variation ranges in fine root depth, biomass, and length density along the RFC gradient showed a decreasing trend in three woody species (Figure 2), which was consistent with our second hypothesis. In these three woody species, fine root depth and biomass increased with growth years (Figures 1 and 2). These results suggest that seedlings are more sensitive to soil heterogeneity than adults, which is consistent with the results of a previous study (Padilla & Pugnaire, 2007). Deepening of the fine roots suggested that older plants were able to access multiple resources and thus adapt to resource constraints with increasing RFC (Li et al., 2020; Padilla & Pugnaire, 2007; Schenk & Jackson, 2005; Zhou et al., 2020).

In addition, soil with 75% RFC aggravated the degree of thinning of fine roots with increasing growth years in all species (Figure 4). The increase in macropores in soil with 75% RFC reduced mechanical resistance, resulting in finer roots with each passing year (Bengough, 2003; Clark et al., 2002; Gargiulo et al., 2015, 2016). Specific fine root length in the soil profile increased with increasing years of growth, and the largest increase was found under 75% RFC (Figure 4). In soils with high RFC, poor resources may be an important reason for long roots (Freschet et al., 2015, 2018). Finer and longer roots facilitate older plants to be in contact with the soil and explore larger soil spaces, which is beneficial for obtaining more resources (Chen et al., 2018; Craine & Dybzinski, 2013; Freschet et al., 2020; Lu et al., 2022).

### 4.3 Fine root vertical profile varied between species

The vertical distribution of fine roots differed significantly between woody and non‐woody species (Figures 1, 2, 3 and 5). Significantly different from the three woody species, in *A. vestita*, the fine root biomass and length density were highly concentrated in the surface soil, especially under 50–75% of RFC (Figures 3, A1, and A2). This result suggests that *A. vestita* tends to rapidly acquire transient resources in shallow soils (Jobbágy & Jackson, 2001; Li et al., 2020; Valladares et al., 2007), such as during occasional rainfall events but might be poor at adapting to long‐term resource limitations. The other three woody species had more roots in deep soils (Figure 1); therefore, they were more resistant to environmental pressure (Schenk & Jackson, 2005; Zhou et al., 2020).

We also found interspecific differences in the degree of variation and vertical profiles of fine roots across RFC gradients and growth years (Figures 1, 2, 3, 4 and A2, A3, A4, A5). The fine root depth of the three woody species showed an increasing trend with an increase in RFCs and growth years (Figures 2 and 4). Woody plants deepen root distribution to obtain resources in resource‐constrained, highly rocky soil (Figures 6 and A6), but this trend became weaker with increasing growth years (Figure 2). In contrast, *A. vestita* showed shallow fine root profiles in soils with high RFC and in older plants (Figures 1 and 2). The results further confirmed that *A. vestita* differs significantly from woody species; it exhibits an opportunistic resource acquisition strategy but has poor resistance to environmental pressure (Jobbágy & Jackson, 2001; Schenk & Jackson, 2005; Valladares et al., 2007; Zhou et al., 2020). Thus, the fine root biomass of *A. vestita* decreased with growth years, whereas that of woody species increased with each passing year (Figure 4).

## 5 CONCLUSION

The results of this study suggest that increases in RFC promote deep distribution of fine roots in *B. brachycarpa*, *S. davidii*, and *C. szechuanensis*, mainly owing to the low water and nutrient levels of the coarse soil and penetration into the deep soil layer. However, increasing years of growth reduced the increase in amplitude in fine root depth and biomass of the three woody species along the RFCs gradient. With increasing growth years, fine root biomass and depth of woody species increased, but the increases in RFCs weaken this cumulative increase year by year. Contrary to the trend of changes in woody plants described above, the non‐woody plants (*A. vestita*) decreased fine root depth and biomass with increasing soil RFCs and growth years. Given the above, woody plants have a strong adaptability to the increase in gravel content, and this ability increases with growth years; although non‐woody plants grow rapidly, they have poor adaptability to high gravel environments for the long term, which indicates that *A. vestita* is only suitable as short‐term pioneer species, while the three woody plants are the best species for long‐term growth when we carry out vegetation restoration in barren areas with high RFCs. These results provide insight into the adaptive processes of plants under rocky soils and have profound management implications for tillage in degraded ecosystems.

## AUTHOR CONTRIBUTIONS

**Hui Hu:** Conceptualization (supporting); data curation (lead); methodology (lead). **Weikai Bao:** Conceptualization (lead); funding acquisition (lead). **Long Huang:** Data curation (supporting); methodology (supporting). **Fanglan Li:** Conceptualization (lead); project administration (lead).

## CONFLICT OF INTEREST STATEMENT

The authors have no conflict of interest to declare.

## Data Availability

Data are publicly available through the Dryad repository (https://doi.org/10.5521/dryad.12311).

## APPENDIX A

**TABLE A1:** Effects of rock fragment content (RFC), years of growth, species and their interaction on *β*, biomass, length density, specific length, diameter, and tissue density of fine root, as revealed by three‐way ANOVAs. (Significant differences between treatment levels in bold).

Parameters	*β* of fine root		Fine root biomass (g m^−2^)		Fine root length density (cm cm^−3^)		Specific fine root length (m g^−1^)		Fine root diameter (mm)		Fine root tissue density (g cm^−3^)	
	*F*	*p*	*F*	*p*	*F*	*p*	*F*	*p*	*F*	*p*	*F*	*p*
RFC	1.15	.333	4.34	**.005**	11.82	**<.001**	13.54	**<.001**	4.11	**.007**	1.01	.387
Growth year	12.63	**<.001**	2.28	.103	50.39	**<.001**	153.67	**<.001**	124.37	**<.001**	18.71	**<.001**
Species	4.94	**.003**	63.44	**<.001**	56.97	**<.001**	13.95	**<.001**	9.64	**<.001**	3.07	**.028**
RFC × Growth year	0.95	.463	2.50	**.022**	4.53	<**.001**	8.21	**<.001**	8.22	**<.001**	0.270	.951
RFC × Species	4.78	**<.001**	3.62	**<.001**	2.89	**.002**	0.77	.646	2.53	**.008**	1.57	.121
Growth year × Species	3.31	**.005**	36.26	**<.001**	22.23	**<.001**	6.79	**<.001**	1.67	.126	4.04	**.001**
RFC × Growth year× Species	0.97	.495	1.74	**.029**	2.22	**.003**	1.06	.390	1.49	.089	1.22	.242

**TABLE A2:** Explains and contribution of soil properties on fine root trait variation in redundancy analysis. See Figure 7 for abbreviations of soil properties.

Soil properties	Explains %	Contribution %	*F*‐value	*p‐*value
SWC	12.0	43.4	64.2	.002
pH	5.6	20.2	33.4	.002
NO_3_‐N	4.2	15.1	23.5	.002
aP	2.6	9.3	16.2	.002
TN	1.6	5.6	9.5	.004
TC	0.9	3.2	5.5	.012
NH_4_‐N	0.6	2.3	4.0	.022
RFC	0.2	0.8	1.4	.222
TP	<0.1	0.1	0.2	.824
DOC	<0.1	<0.1	<0.1	.954
